# Professional Education: A Criticism

**Published:** 1902-03

**Authors:** 


### Editorial.

### PROFESSIONAL EDUCATION: A CRITICISM.

The subject of professional training, and especially preliminary education, has claimed the serious attention of many in this country, and has become an important topic for discussion in the older civilizations, if we may judge by the recent publications of the proceedings of the International Dental Federation recently held in England. The discussion there was mainly confined to the professional side, and only included incidentally the question of preliminary training. That the extended consideration of the subject in all its aspects is bound to prove of great value needs no argument, being a self-evident fact. The wider the knowledge contributed of the methods of education in force in various countries, the closer will be the approximation to a harmonious development everywhere, resulting in the establishment of an educational equilibrium not only in general training, but in our own specialty, modifying and improving the dental curricula of all countries. That this will be speedily accomplished is not to be expected, nor would it be desirable. All radical changes are to be deprecated. It is possible also that there must always be a decided difference in methods, while it may equally be possible to reach the same result.

In this country it cannot be truthfully stated that a system of general education exists. We have our public school system, which varies with different States and in different localities in the same State. In some it is worthy the highest praise; in others it is not above a very ordinary standard. There is no such interchangeable system here as in Continental Europe, carried to great perfection in Germany, nor would such a system be possible covering the entire United States. It is, therefore, useless to expect uniformity of methods, and yet this wide diversity produces results nearly equal to the more systematic training. The criticism made frequently by foreigners who have had but a few weeks or months of observation are of no value. Their standard of observation is defective, inasmuch as they are unable to appreciate any method not in harmony with that to which they have been accustomed.

The object of this article is not so much to treat of education as an entirety, or even to attempt to cover in part the subject so ably discussed by trained minds at home and abroad, but to call attention to some of the errors of statement and erroneous conclusions based upon defective premises in their line of argument.

It is unfortunate that many of those who attempt the discussion of this topic are, by training, one-sided men, some viewing from the purely scholastic side, others from that conjoined with the medical, and still others unite these and add a third, the dental. It is, perhaps, impossible to erect a platform upon which all of these elements can harmoniously stand. Time alone can effect changes from this chaotic mental condition; and it would, perhaps, be a waste of energy to continue the discussion, were it not for the fact that all mental energy is equally valuable in the individual and in the mass, resolving complexity into simplicity and antagonisms into harmony, thus bringing about stable views on all disputed subjects.

In the present number our readers will find an able treatise on this subject read before the American Academy of Dental Science, Boston, by Professor Macvane, of Harvard University. He very naturally views the subject from the stand-point of the scholar; but, while a professor of history, he has been unfortunate in this paper in some of his statements, and equally so as to some of his supposed facts.

It is difficult to understand the thoughts he wishes to convey in the following quotation: "Professional education . . . is a new one in the world." He certainly cannot mean by the word *new* that professional education is modern. It is possible he refers to medical-college training as deserving that title, yet during the Arabic period in the history of medicine, A.D. 640, "students from all parts flocked to the academies." Perhaps he proposes to date professional education in this country from the founding of the Department of Medicine in the University of Pennsylvania, in 1765, or the organization of the Medical Department of King's College (now Columbia), New York, 1767, or of Harvard Department of Medicine in 1783. He does not, however, explain his meaning, and the reader is left in greater mental confusion by the following sentence: "Provision for professional training came later than the Civil War." From what has already been shown, professional training was of very respectable age in all the subjects of law, medicine, and dentistry when the Civil War was opened at Fort Sumter.

The writer is unquestionably correct in his statement that "the demand for it [the reduction of the college course to three years] has come from some of the professional schools." This has been a natural demand to avoid extending the scholastic training beyond a certain age. Farther on he states that "young men do not get the proper degree of maturity for real university work until they get to be twenty-one years of age or so." This means, of course, that the student of medicine will have reached twenty-five before graduation in medicine, and adding another year of hospital work and an additional six to ten years spent in efforts to build up a practice, and the most active portion of the life has departed.

The essayist asks, "Is it not evidently of more importance to have well-trained men, citizens with trained minds, than to have the younger members of the professions very highly trained at the very moment of their entrance into the profession (practice)? Is not the young dentist bound, in any case, to be more or less of a journeyman for some years?" The reply to this is that it is certainly desirable to have well-trained minds, but where there might be a difference of opinion would be in the mental training which is best adapted for the cultivation of the mind. It must be self-evident that no one single line of study will effectually accomplish this object. If it be assumed that neither medical nor dental training has a part in such education, then the writer must perforce part company with the essayist. There may be different degrees of value in study, but all mental work that requires careful analysis is an educational force. The young dentist when he graduates is not supposed to be a journeyman. The aim of all dental education is to make him a well-rounded practitioner when he receives his diploma. He has had, in his three years of constant study and practice, all the possibilities to meet that will come to any advanced practitioner. He may lack, it is true, that maturity of thought that can only be acquired by a lifetime of practice. The college that would send out a mere journeyman to acquire skill through experimentation upon patients would deserve, and certainly would receive, merited censure.

It is with regret that the author of this paper has subjected himself to friendly criticism, but it is essential that one occupying his position should seek authoritative sources for his historical information. If we may judge from the subjoined quotation, this he has not done, for he writes: "Of your own dental schools, three have no express admission requirements. Eighteen have purely grammar school subjects for entrance. Another eighteen seem to require about a first year in a high-school course. . . . Eleven others have requirements that would be covered by about two years of the ordinary high school courses, . . . and six of the best may be fairly said to require about three years of a high school course." In view of the fact that the regulations governing dental colleges in this country could have been readily discovered, it would seem that such errors of statement should not have been made. The essayist could have learned that the fifty-two dental colleges of this country, members of the National Association of Dental Faculties, are required, under rather severe penalties, to comply with the rules of said Association and to live up to the standard it has established. Any of the colleges may go beyond this requirement, but cannot go below. The entrance standard has been gradually raised from graduation at grammar schools to the present standard. The following rule, taken from the annual report of this body, meeting at Milwaukee, August, 1901, will, it is presumed, fully explain the position occupied by this organization:

"The minimum preliminary educational requirement of colleges of this Association, beginning with the session of 1902-1903, shall be a certificate of entrance into the third year of a high school, or its equivalent. The preliminary examination to be placed in the hands of the State Superintendent of Public Instruction.

"Nothing in this rule shall be construed to interfere with colleges of this Association that are able to maintain a higher standard of preliminary education."

The writer has not made these criticisms in any captious spirit. He recognizes the difficulty an essayist must meet when he attempts an entrance to the domain of another and quite distinct calling, but this fact emphasizes the importance of a broad comprehension of all educational methods before attempting an analysis of any single effort.

It is hoped that the wide discussion given this very important subject will create an increased interest in dental educational work, and result in an equalization of all the varying degrees of training the world over, so that eventually the young dentist everywhere will take rank, each with the other, no matter where educated, and at the same time hold a scholastic and professional position beyond criticism.

